# Downregulation of Purkinje Cell Activity by Modulators of Small Conductance Calcium-Activated Potassium Channels In Rat Cerebellum

**Published:** 2016

**Authors:** T. V. Karelina, Yu. D. Stepanenko, P. A. Abushik, D. A. Sibarov, S. M. Antonov

**Affiliations:** I.M. Sechenov Institute of Evolutionary Physiology and Biochemistry of the Russian Academy of Sciences, prosp. Toreza, 44, Saint-Petersburg, 194223, Russia

**Keywords:** cerebellum, Purkinje cells, SK channels, ageing

## Abstract

Small-conductance calcium-activated potassium channels (SK channels) are widely expressed in CNS tissues. Their functions, however, have not been well studied. Participation of SK channels in Purkinje cell (PC) pacemaker activity has been studied predominantly *in vitro*. Here we studied for the first time the effects of SK channel activation by NS309 or CyPPA on the PC simple spike frequency *in vivo *in adult (3 – 6 months) and aged (22 – 28 months) rats using extracellular microelectrode recordings. Both pharmacological agents caused a statistically significant decrease in the PC simple spike frequency. The maximum value of the decrease in the simple spike frequency did not depend on age, whereas a statistically significant inhibition of the spike frequency was achieved faster in aged animals than in adult ones. In experiments on cultured neurons PCs were identified by the expression of calbindin as the PC-specific marker. Registration of transmembrane currents in cerebellar neurons revealed the direct action of NS309 and CyPPA on the SK channels of PC consisted in the enhancement of outward potassium currents and action potential after-hyperpolarization. Thus, SK channel activators can compensate for age-related changes of the autorhythmic functions of the cerebellum.

## INTRODUCTION

The cerebellum is an important part of the CNS due to the variety of functions it performs. The cerebellum plays a key role in motor activity by monitoring all motor acts and minimizing the error between an intended and performed action [1]. One of the manifestations of cerebellar dysfunction is spinocerebellar ataxia, a disruption of the accuracy and coordination of voluntary movements, the development of which is often associated with the death or dysfunction of Purkinje cells (PC). In addition, the dysfunction manifested in the change in the pattern of PC activity can occur prior to the disruption of motor activity. For example, in mice with genetically determined hereditary spinocerebellar ataxia type 2, physical activity begins to deteriorate on the 8th week, the number of PC start to decrease on the 12th week, while the decrease in the PC discharge frequency can be registered as early as on week 6 of postnatal development [2]. Studies carried out on sections of the cerebellum of mice and rats have demonstrated that pacemaker activity changes in such neurodegenerative diseases as spinocerebellar ataxia type 2 and 3, as well as episodic ataxia type 2 [3-5].

Ca^2+^-activated K+ channels are expressed by many neurons of the CNS and are of three types: channels of large (BK), small (SK), and intermediate (IK) conductance [6]. SK channels are voltage-independent channels which are directly activated only by Ca^2+^ at submicromolar concentrations [7], enhancing the action potential after-hyperpolarization in neurons [6]. PCs are characterized by a pronounced expression of small-conductance Ca^2+^-activated K^+^ channels of the SK2 subtype [8]. Blocking SK2 channels with apamin in PCs that have a trimodal pattern of activity causes a shortening of its cycles, while in cells with a tonic type of discharge this leads to increased frequency and occurrence of explosive discharge [9].

We have previously shown that the frequency of simple spikes increases and the depression period after a complex spike decreases in normal aging in PCs [10, 11]. A similar age-related increase in the frequency of PC discharges was shown in the study by Kasumu et al. in mutant mice with a model of spinocerebellar ataxia type 2 [12].

Almost all studies of SK channels in cerebellar PC are performed under *in vitro *conditions. However, in this case, the integrity of the cerebellum structure itself, as well as the afferent and interneuronal connections, is disturbed. Numerous papers devoted to genetically predetermined cerebellar pathologies have been based mainly on young and adult animals, whereas information on the changes in PC functions in aged animals during normal aging remains scant. For this reason, a comparative study of the features of PC function *in vivo *in late ontogenesis appears to be important. Taking into account the direct involvement of SK channels in the regulation of the pattern of cerebellar PC activity and its change during aging, as well as the contribution of SK channels to the development of various neurodegenerative diseases, the objective of this work was to perform a comparative study of the contribution of SK channels to the pattern of PC activity in adult and aged rats.

## EXPERIMENTAL SECTION

**Extracellular registration of cerebellar PC activity *in vivo***

**Fig. 1:**
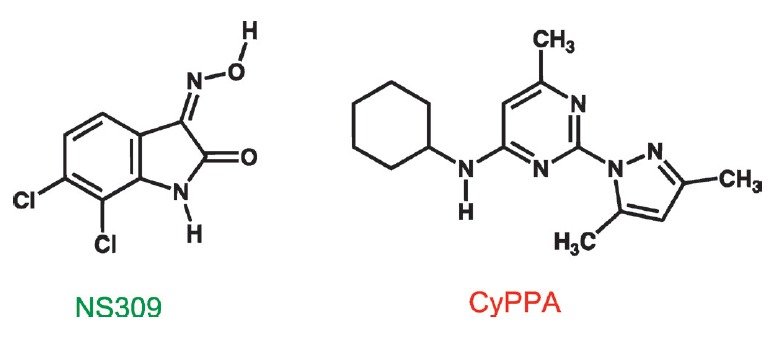
Chemical structure of the activators, or allosteric modulators, of SK channels that increase their sensitivity to calcium

This study was conducted on Wistar rat males and females. The animals were divided into two groups during the experiment: adults (3 to 6 months) and aged (21 to 24 months). For the anesthesia of the animals, urethane was used, which was administered intraperitoneally at a rate of 1,300 and 1,000 mg/kg of body weight of the adult and aged rats, respectively. PCs were registered and identified as previously described [13]. In anesthetized animals, the scalp and muscle layer were removed, and a hole 1 mm in diameter was drilled in the occipital bone above the cerebellar vermian. Next, the animal was fixed in a stereotaxic frame. For the registration of extracellular PC activity, microelectrodes made of borosilicate glass (outer diameter: 1.5 mm, inner diameter: 1.10 mm, Sutter Instrument, USA) and filled with a solution of 2.5 M NaCl were used. A microelectrode was placed in the cerebellar tissue using an automatic manipulator with a penetration step of 5 μm and a depth of up to 5 mm. PCs were identified by their characteristic pattern of activity: the presence of simple and complex spikes, as well as a pause after a complex spike before a series of simple spikes. The signal of the registered cell was amplified (AC/DC Differential Amplifier, model 3000, A-M Systems, Inc., USA) and digitized at a sampling frequency of 10,000 smp/s (ADC L-791, “L-CARD” Ltd, Russia) using the original Bioactivity Recorder v.5.3 software developed by D.A. Sibarov [http://sibarov.ru/ index.php?slab=software] for further analysis of simple frequency spikes using the Clampfit 10.2 software (Molecular Devices Corp., USA). Active substances were loaded according to the standard method [14] by application to the exposed surface of the cerebellum in the area of microelectrode placement. First, control series of experiments were conducted in which the application of saline (0.9% NaCl) was performed. Then, a positive modulator of SK and IK channels NS309 (Tocris, USA) and selective activator of small-conductance calcium-activated potassium channels of the SK2 and SK3 subtypes, CyPPA (Tocris, USA), were used in the next series of experiments (*Fig. 1*). The concentrations of active substances chosen depending on the depth of microelectrode penetration were 100–200 μM for NS309 and 1–2 mM for CyPPA. Solutions of the active substances were prepared in 0.9% NaCl. Registration of PC activity was performed for 30 sec prior to substance application and then during periods of the same duration 5, 15, 30, 45 and 60 min after application.

In order to compare the control and experimental data, the frequency of simple spikes was measured in each cell for 30 sec for all the specified time points. Then, relative frequencies for each time point were calculated taking the frequency of spikes prior to application as the unit rate. The mean frequency value and standard error of mean (SEM) were calculated for each time point. Two-way ANOVA with Bonferroni’s post-test was used for the assessment of the statistical significance of the differences between the control and experimental data, i. e. under the action of positive modulators. For a comparison of the original frequency with the average values at each time point in the control series of experiments, one-way ANOVA was used.

**Preparation of the primary culture of cerebellar neurons**

In primary cultures of neurons isolated from different parts of the embryonic brain, particularly the cerebral cortex [15] and the cerebellum [16], a differentiation of the main types of neurons characteristic of these regions in the adult brain takes place. For example, a primary culture of cerebellar neurons has been successfully used to study the properties of Purkinje cells [17]. Primary cultures were prepared from the cerebellum of embryos on day 20–21 of prenatal development (E20–E21). In order to obtain a suspension of cerebellum cells, the isolated tissue was placed in a trypsin solution (0.04 mg/ml) and the cells were then treated with a DNase solution (0.04 mg/ml), trypsin inhibitor, and fetal bovine serum. After centrifugation, cell dissociation was performed by pipetting in the medium. Dispersed cells were cultured on 7-mm glasses treated with a poly-*D*-lysine in Neurobasal medium (Gibco, USA) supplemented with B27 (Gibco, USA), *L*-glutamine (Gibco, USA), and 20 mM KCl to increase the chance of survival of cerebellar neurons, including Purkinje cells and granular neurons [18].

**Registration of neuronal currents by the voltage-clamp method**

The direct effect of SK channel modulators on neurons in a primary culture of cerebellar neurons was confirmed using the voltage-clamp in the whole-cell configuration. In the current fixation mode, we registered the action potential shape, and in the voltage-clamp mode, we obtained the current-to-voltage characteristics of SK channels.

Experiments in cerebellar culture cells were performed on day 7–8 *in vitro *(DIV 7–8). Extracellular saline of the following composition was used in the experiments: 140 mM NaCl, 2.8 mM KCl, 2 mM CaCl, 10 mM HEPES. Intracellular solution for microelectrode filling had the following composition (mM): 9 NaCl, 17.5 KCl, 121.5 K-gluconate, 1 MgCl_2_, 10 HEPES, 0.2 EGTA, 2 MgATP, 0.5 NaGTP. A MultiClamp 700B amplifier with a Digidata 1440A data collection system and the pClamp v10.2 software (Molecular Devices, USA) were used for current registration. The sampling frequency was 20,000 smp/s. The initially registered signal was subjected to preliminary analog filtering (equivalent of the 8th-order Bessel high-pass filter) with a cutoff frequency of 200 Hz. For the application of test substances (100 μM CyPPA or 10 μM NS309), a BPS-4-based system for a quick change of solutions (Ala Scientific Instruments, USA) with a multi-channel capillary perfusion was used, the tip of which was placed 200–300 μm away from the registered cell. PCs were identified by soma size, which is significantly larger than for other types of neurons (about 4-fold) and rhythmic generation of action potentials.

The current-to-voltage characteristics of the channels activated by CyPPA and NS309 were determined based on the difference between the neuronal currents registered under the Ramp protocol: a smooth change in the potential from –100 to +60 mV in 5 seconds before and after the application of substances. The statistical significance of the changes in the after-hyperpolarization of spikes under the influence of positive modulators of SK and IK channels was evaluated using the unpaired Student’s *t*-test.

**Immunohistochemical staining of Purkinje cells**

The presence of PC in a primary culture of rat cerebellar cells was additionally monitored using an immunocytochemistry analysis of calbindin protein expression, a marker of this type of neurons [19]. During preparation for immunocytochemical staining, the glasses with cells were fixed with a 4% formaldehyde solution and then treated with ammonium chloride (0.535 mg/ml), Triton X-100 (0.2% solution), and glycine (15 mg/ml). Non-specific binding of antibodies was blocked by treating the glasses with cells with a 2% solution of bovine serum albumin. All solutions were prepared based on PBS. For the identification of calbindin in PC, mouse primary monoclonal antibodies to this protein (Calbindin-D28k, Abcam, ab82812) were used. Immunopositive reaction was visualized using secondary antibodies conjugated to Alexa 633 fluorochrome (Molecular Probes A21052, Life Technologies, USA). In order to avoid rapid bleaching of fluorescent dyes, the glasses, treated with antibodies, were fixed on slides with an adhesive containing the Mowiol compound (Sigma-Aldrich, Germany). The fluorescence of immunopositive neurons was registered on a confocal scanning microscope Leica SP5 MP (Leica Microsystems Inc., Germany) equipped with ×63 oil immersion lens (HCX APO CS 63×/1.4; Leica Microsystems, Inc., Germany). Excitation of the Alexa 633 dye was performed using an argon laser with a wavelength of 633 nm. The dye emission range was 640–700 nm. Image processing was performed using the Leica LAS AF software (Leica Microsystems Inc., Germany).

## RESULTS

**Influence of positive modulators of SK channels on the PC simple spike frequency in aged rats**

**Fig. 2:**
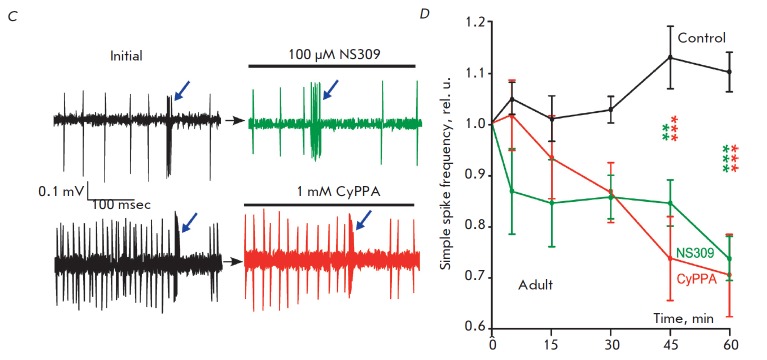
Activity of rat cerebellar Purkinje cells under the influence of positive modulators of calcium-activated potassium channels. *A *– aged rats. Fragments of a recording of the activity of individual PCs prior to and after CyPPA and NS309 application. Arrows indicate episodes of occurrence of complex spikes. *B *– aged rats. Change in the medium frequency of simple spikes of cerebellar PC for 1 hour after the application of saline or SK channel activators. *C *– adult rats. Fragments of a recording of the activity of individual PCs prior to and after CyPPA and NS309 application. *D *– adult rats. Change in the medium frequency of simple spikes of cerebellar PC for 1 hour after the application of saline or SK channel activators. Green asterisks indicate a statistically significant difference from the control frequency values at the corresponding moments of NS309 application, red – CyPPA application (ANOVA, Bonferroni post-test * – *p* < 0.05, ** – *p* < 0.01, *** – *p* < 0.001)

In our study, all afferent PC connections were preserved during the registration of PC action potentials *in vivo*, in contrast to the experiments carried out on the tissue sections, which caused irregular pulse intervals. Examples of a typical PC activity in aged animals are shown in the control and upon NS309 action, as well as in the control and upon CyPPA action (*Fig. 2A*). Application of physiological saline did not cause significant changes in the frequency of PC simple spikes in aged animals for 60 minutes (15 to 43 Hz at the beginning and 16 to 48 Hz at the end, *p *> 0.95, *n *= 7, ANOVA). The increase in the average value of the relative frequency of simple spikes at certain periods of registration was 0–3% and reached its maximum value 30 minutes after application (*Fig. 2B*). These results clearly demonstrate that the application procedure did not affect the pattern of PC discharge.

NS309 caused a gradual decrease in the simple spike frequency of PCs. Significant differences in the frequency in the control at the appropriate time point (*p * < 0.05, *n *= 10, ANOVA, Bonferroni post-test) were revealed 15 min after application, which persisted until the end of the registration period. The lowest value of the simple spike frequency was achieved 60 min after the start of application and was on average 29% lower than the control value at this time (*Fig. 2B*).

Upon CyPPA action, a statistically significant decrease in the simple spike frequency of the PC discharge (*p * < 0.001, *n *= 11, ANOVA, Bonferroni post-test) occurred 30 minutes after the start of registration, i. e. later than in the case of NS309, while the maximum decrease at the end of registration was on average 21% (*Fig. 2B*).

**Influence of positive modulators of SK channels on the PC simple spike frequency in aged rats**

*Figure 2B* shows examples of a typical PC activity in adult animals in the control and under NS309 action, as well as in the control and under CyPPA action. In this age group, a tendency for an increase in the frequency of simple spikes was noted upon the application of saline. However, these changes were not statistically significant (*p* > 0.10, *n* = 8, ANOVA).

In adult animals, a decrease in the simple spike frequency of the PC discharge was achieved 45 min after the start of NS309 application (*p* < 0.001, *n *= 9, ANOVA, Bonferroni post-test), i. e. later than in aged mice. The maximum, 33% on average, decrease occurred at the end of the 60th minute of registration (*Fig. 2D*).

Upon CyPPA application, as in the case of NS309, a statistically significant decrease in the simple spike frequency of the PC discharge was achieved 45 minutes after the start (*p * < 0.001, *n *= 8, ANOVA, Bonferroni post-test). The maximum decrease occurred after 60 minutes (a median 36% decrease) (*Fig. 2D*).

Despite the fact that a significant decrease in the simple spike frequency upon the action of both positive modulators of SK channels was achieved earlier in aged rats compared to the control, the maximum effect of CyPPA (*n *= 11) and NS309 (*n *= 10) did not differ in the animals regardless of age. Moreover, we did not manage to detect a statistically significant difference in the effects of NS309 and CyPPA in aged and adult rats (*p *> 0.8, ANOVA).

**Electrical activity of cerebellar neurons in culture under the influence of SK channel modulators**

**Fig. 3:**
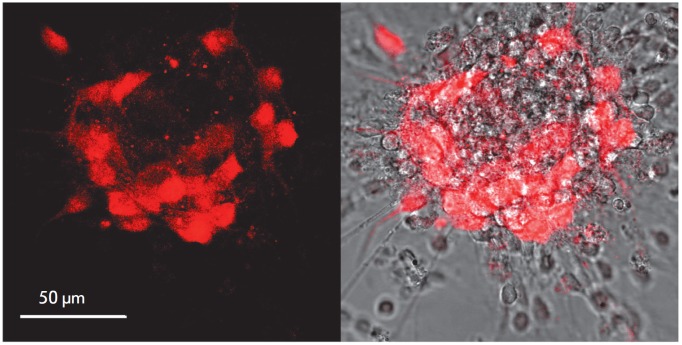
Purkinje cells in a DIV 7 primary culture of rat cerebellum neurons. *A *– fluorescent image of the immunopositive response to the calbindin protein, which is expressed only in Purkinje cells. *B *– result of image superposition in transmitted light and fluorescence of the calbindin-D28k protein obtained by immunolabeling of the corresponding protein with antibodies conjugated with Alexa 633 fluorochrome

Cerebellar cells in a primary culture form a neuronal network on day 7 of culturing, wherein immunohistochemical staining for calbindin-D28k confirmed the presence of Purkinje cells distinguished by the large size of their soma (*Fig. 3*). In an electrophysiological study, part of the “large” neurons were characterized by a spontaneous periodic generation of an action potential (AP) typical of Purkinje cells.

**Fig. 4:**
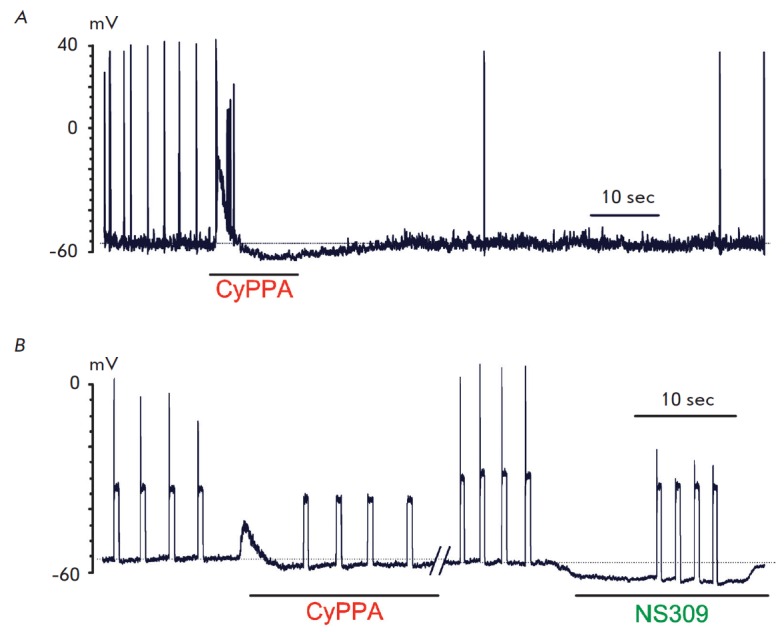
Examples of the influence of CyPPA and NS309 on the spontaneous generation (*A*) and action potentials caused by a depolarizing stimulus (*B*) in cerebellar neurons. Intracellular registration of the membrane potential in the current-clamp mode. Action potentials were evoked by current injection (1.1 threshold value) through a recording electrode

**Fig. 5:**
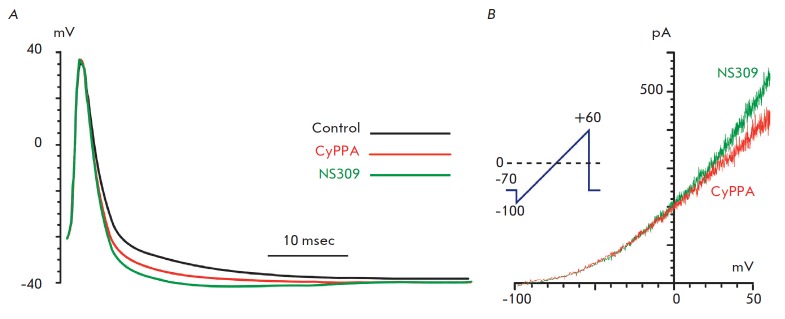
Influence of CyPPA and NS309 on the after-hyperpolarization in the neurons of the cerebellum. *A *– mean action potentials in the control and in the presence of 100 μM CyPPA or 10 μM NS309 (no less than 140 AP was averaged for each condition) registered upon fixation of a neuronal current. *B *– current-voltage characteristics of the channels activated by CyPPA and NS309 in cerebellar neurons registered in the current-clamp mode. Insertion illustrates the Ramp protocol used to measure the current-to-voltage characteristics of SK channels

It cannot be unambiguously concluded from the *in vivo *experiments whether the studied modulators act directly on PC or whether their effect is mediated by net interactions through influence on intercalary neurons. In order to test the hypothesis on the direct action of CyPPA and NS309 on cerebellar neurons, we studied their impact on spike generation by neurons in a primary culture using local perfusion. In neurons with spontaneous generation of AP (*Fig. 4A*), the application of 10 μM CyPPA caused transient depolarization followed by hyperpolarization, accompanied by an attenuation of the spontaneous generation of AP (*n *= 5). The generation of spikes caused by the injection of a current of threshold amplitude in neurons without spontaneous activity was effectively suppressed by the application of both 100 μM CyPPA and 10 μM NS309 (*n *= 18, *Fig. 4B*). Both substances induced hyperpolarization, with the effect being more pronounced in the case of NS309. A comparison of the forms of AP of a spontaneously active neuron before and after the application of potassium channel activators showed that both substances enhance AP after-hyperpolarization (*Fig. 5A*). Such phenomenology is typical of the activation of SK channels. In addition, after-hyperpolarization at the point of minimum was increased by 3.1 ± 0.3 mV upon the action of CyPPA and by 6.1 ± 0.3 mV upon the action of NS309. The effect of NS309 was significantly stronger than that of CyPPA (*n *= 140; *p * < 0.01, unpaired Student’s *t*-test). The current-to-voltage characteristics of the channels activated by CyPPA and NS309 presented in *Fig. 5B* are also typical of SK channels [20].

Thus, CyPPA and NS309 similarly suppress the generation of AP by Purkinje cells both *in vivo *when applied to the cerebellum surface and *in vitro *in a primary culture of neurons.

## DISCUSSION

**Fig. 6:**
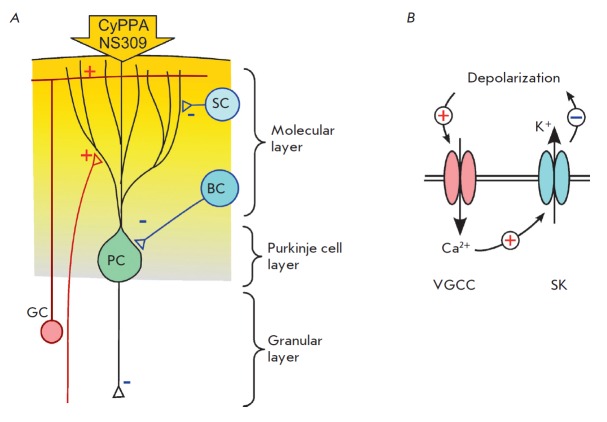
The scheme of SK channel modulator action on Purkinje cells (PC) upon application to the surface of the cerebellum. *A *– simplified diagram of neuronal connections of PC in the cerebellar cortex. (+) – excitatory and (–) – inhibitory synaptic connections, PC – Purkinje cell, SC – stellate cell, BC – basket cell, GC – granule cell. *B *–relationships between the key ion channels of the PC regulating their autorhythmic activity

There is evidence indicating that NS309 and CyPPA, positive modulators of SK channels, change the pattern of neuronal activity. Experiments performed on sections of the cerebellum have demonstrated a decrease in the frequency of PC discharge after NS309 application in a bath with a washing solution [3]. Similar results were obtained in *in vivo *experiments, showing that the use of CyPPA and NS309 causes a decrease in the discharge frequency of substantia nigra dopaminergic neurons [21, 22]. In our experiments conducted *in vitro *on primary cultures of cerebellar neurons, CyPPA and NS309 also effectively suppressed the generation of spontaneous and evoked spikes (*Fig. 4A,B*) because of an increase in the after-hyperpolarization (*Fig. 5A*) caused by SK channel activation (*Fig. 5B*) directly in the studied neurons. CyPPA is a selective activator of SK2 and SK3 channels, while only Purkinje cells are characterized by a high expression of SK2 in the cerebellar cortex in the late prenatal and postnatal periods [8], which makes these cells the primary target of the action of SK channel activators. Due to the anatomical structure of the cerebellum, activators of SK channels, when applied to the surface, first penetrate the molecular layer, where they can interact with the dendritic tree of PC and PC somas during diffusion (*Fig. 6A*). The roles of dendritic and somatic SK channels vary. In young rats (10–90 days of age), a blockage of somatic SK channels enhances the frequency of autorhythmic activity of PC, whereas a blockage of dendritic SK channels not only enhances the frequency of simple spikes, but also reduces the leakage current and improves transmission in synaptic inputs to PC [9]. Furthermore, a blockage of only dendritic SK channels had a significantly lesser effect than a blockage of dendritic and somatic SK channels [9]. Apparently, in our experiments, the gradual diffusion of NS309 or CyPPA from PC dendrites toward the soma determines the gradual enhancement of the effect of these SK channel activators with time. In our experiments conducted *in vivo*, activation of SK channels by the positive modulators CyPPA and NS309 resulted in a change in the pattern of PC activity. In both the groups of adult and aged rats, the simple spike frequency was statistically significantly reduced compared to the control series. Although the reduction in the simple spike frequency caused by SK channel modulators was almost identical for the groups of adult and aged rats, the decrease in the frequency of simple spikes in aged rats occurred earlier compared to the control: 30 min after CyPPA application and 15 min after NS309 application. In adult rats, the decrease in the simple spike frequency under the influence of both substances was observed after 45 min.

CyPPA and NS309 in saturating concentrations enhance the sensitivity of SK channels to intracellular calcium many-fold, resulting in the fact that maximum activation of these channels is achieved at any physiological concentration of intracellular calcium [23]. In our experiments, SK channel activators achieved saturating concentrations near PC gradually during diffusion. Thus, the rate of the effect’s onset could depend on the age-related features of the dynamics of intracellular calcium. Activation of SK channels in PC is determined by the entry of calcium through voltage-gated calcium channels (VGCC) (*Fig. 6B*). Reduced Cav2.1 (P/Q-type VGCC) expression and almost complete knockout of Cav1.3 (L-type VGCC) is noted in the molecular layer of the cerebellum of aged animals [24]. Cav1.3 is activated at very low values of membrane potential; i. e., it is the most sensitive to depolarization [25], whereas a deficiency of Cav2.1 can lead to ataxia type 2 [26-28], since a large proportion of the calcium in PC entering through VGCC is accounted for by Cav2.1 [29]. Under conditions of age-related partial loss of VGCC and the calcium signal associated with it, the normal activation of SK channels can be affected. Moreover, oscillations of membranous intracellular calcium modulate the activity of other types of receptors; in particular, desensitization of glutamate receptors, which provide glutamatergic synaptic transmission [30].

The study carried out *in vivo *in adult mice showed that the simple spike frequency of PC discharge upon NS309 application is reduced much more than in CyPPA application [13]. The results presented in the current paper demonstrate a similar decrease in the simple spike frequency under the influence of positive modulators of SK channels, NS309 and CyPPA, in adult rats. A tendency to a more pronounced decrease in the simple spike frequency was noted under the action of NS309 rather than CyPPA in the group of aged animals. Moreover, the decrease in the simple spike frequency after CyPPA application was comparable with earlier findings in mice, while NS309 had a more pronounced influence on the activity of cerebellar PC in mice than rats. CyPPA is known to be a selective modulator of SK channels, while NS309 is also an activator of the IK channels [31] expressed in PC dendrites, where they modulate temporal summation of synaptic inputs [32]. Thus, in our experiments *in vitro*, NS309 enhanced the after-hyperpolarization of PC more so than CyPPA. Apparently, due to this, a decrease in the simple spike frequency in PC is more pronounced upon NS309 action than in the case of CyPPA, which activates only SK2 channels in PC. The difference in the efficiency of the NS309 effect on the simple spike frequency of cerebellar PC discharge of mice and rats is probably due to the species differences in the expression and function of IK channel features in the cerebellum or the features of the diffusion barriers upon the specific method of application of these substances in these animals.

Application of positive modulators of SK channels in animals that serve as a model of some types of spinocerebellar ataxias leads to the restoration of an impaired pattern of PC activity and, in some cases, to the disappearance of ataxia symptoms [4, 5, 8, 9], which implies that they have a therapeutic effect. Moreover, the use of SK channel activators allows one to compensate for the age-related changes in the autorhythmic functions of cerebellar PC. The reasons for the age-related differences in the effects of SK channel activators require further study, since they could be associated to a deficit in the functions of the voltage-gated calcium channels of PC.

## Abbreviations

PC: Purkinje cells
calbindin: calbindin-D28k
CyPPA: N-cyclohexyl-N-[2-(3,5-dimethylpyrazol-1-yl)-6-methyl-4-pyrimidinamine
NS309: 6,7-dichloro-1H-indole-2,3-dione 3-oxime
SK channels: small-conductance Ca2+-activated K+ channels
VGCC: voltage-gated calcium channels
